# Combined Effects of 1-MCP and Modified Atmosphere Packaging on Flavor Quality and Volatile Profile of Cold-Stored Strawberries Revealed by Untargeted GC-MS Analysis

**DOI:** 10.3390/foods14172936

**Published:** 2025-08-22

**Authors:** Yukang Gu, Minghui Xu, Jun Liu, Juan Kan, Man Zhang, Lixia Xiao, Xiaodong Yang, Xiaohua Qi, Chunlu Qian

**Affiliations:** 1 Department of Food Science and Engineering, School of Food Science and Engineering, Yangzhou University, Yangzhou 225127, China; 15161393137@163.com (Y.G.); mhui0427@163.com (M.X.); junliu@yzu.edu.cn (J.L.); kanjuan@yzu.edu.cn (J.K.); mzhang@yzu.edu.cn (M.Z.); lxxiao@yzu.edu.cn (L.X.); 2 Department of Horticulture, College of Horticulture and Landscape Architecture, Yangzhou University, Yangzhou 225009, China; yxd@yzu.edu.cn (X.Y.); xhqi@yzu.edu.cn (X.Q.)

**Keywords:** relative odor activity value, enzyme activities, sensory acceptability, key characteristic aroma compounds

## Abstract

Strawberries are highly perishable despite their popularity, as their limited shelf life compromises both freshness and market value. The study investigated the effects of 1-methylcyclopropene (1-MCP), modified atmosphere packaging (MAP), and their combined treatments on the quality and flavor of strawberries during cold storage and simulated shelf life. 1-MCP was applied by enclosing strawberry fruits in a hermetically sealed container and exposing them to 250 nL/L 1-MCP at 20 °C for 18 h. Three initial MAP gas compositions were tested: MAP1 (5% O_2_, 15% CO_2_, 80% N_2_), MAP2 (10% O_2_, 10% CO_2_, 80% N_2_), and MAP3 (15% O_2_, 5% CO_2_, 80% N_2_), with MAP1 identified as optimal based on strawberry postharvest quality metrics. The results showed that all treatments could inhibit the deterioration of strawberry quality, and the 1-MCP + MAP treatment had the best fresh-keeping effect. Untargeted Gas Chromatography-Mass Spectrometry (GC-MS) analysis identified 85 volatile compounds, and sensory correlation analysis revealed that 1-MCP + MAP-treated strawberries maintained the highest consumer acceptability, with odor characteristics closely resembling those of pre-storage controls. Further studies demonstrated that the combined treatment uniquely suppressed the generation of fatty acid oxidation-derived volatiles while stabilizing critical aroma-active esters, thereby decelerating flavor degradation. Collectively, these findings highlight the potential of 1-MCP + MAP as a postharvest strategy to delay the postharvest senescence of strawberries and maintain their storage quality. GC-MS provided a scientific method for the flavor quality evaluation of this preservation technology.

## 1. Introduction

Strawberry (*Fragaria x ananassa* Duch.) is one of the most popular fruits globally. They are typical berries with delicate fruit peels and tender textures when they reach full ripeness [1]. Strawberries are non-climacteric fruits, distinct from climacteric ones such as apples and bananas. They display low postharvest respiration rates and ethylene production. Despite their low sensitivity to ethylene, exogenous or ambient trace ethylene can still impact postharvest quality by regulating cell membrane permeability and accelerating oxidative metabolism [2]. Commercially harvested strawberries have a fragrant and sweet flavor, but they are perishable after harvest and their flavor quality is prone to change during storage. Cold storage is the most common method for preserving strawberries. Pretreatment including hot air, modified atmosphere packaging (MAP) [3], nitric oxide, and 1-methylcyclopropene (1-MCP) [4,5] can effectively improve the quality of strawberries during cold storage by regulating physiological processes, inhibiting senescence-related reactions, and maintaining sensory and nutritional qualities.

Food flavor is a key factor influencing consumers’ preferences and purchasing choices [6], and its formation mechanism is complex, involving volatile substances, non-volatile substances, and macromolecular compounds [7]. This sensory experience stems from the combined effect of the chemosensory system. It is mainly the dual stimulation of smell and taste triggered by chemical substances contained in food raw materials themselves or generated during the processing and storage [8].

1-MCP, a potent ethylene inhibitor, is commonly applied to extend fruit shelf life [9,10] and could enhance strawberry quality under refrigeration [11,12]. MAP effectively lowers fruit respiration and metabolic rates by modifying packaging gas composition [3]. The combination of treatments can also reduce the decay of strawberries during storage [13,14].

The flavor quality of strawberries is composed of various volatile substances [15]. More than 360 volatile compounds have been found in strawberries [16], but only a few contribute to the overall flavor. Compounds such as hexanoic acid, methyl and ethyl esters [17], linalool, nerolidol, C6 aldehydes, γ-dodecalactone, benzaldehyde, and 4-methoxy-2,5-dimethyl-(mesifuran) are characteristic flavor components of strawberries [18,19]. The flavor quality of strawberries varies with the cultivar and fruit formation conditions [20], but limited information about the change pattern of its flavor quality is available.

Gas Chromatography-Mass Spectrometry (GC-MS) is an analytical technique that integrates the high-resolution separation capability of GC with the high-sensitivity detection and structural elucidation capabilities of MS [21,22]. This hyphenated methodology finds widespread application in the identification and quantitative determination of volatile and semi-volatile organic compounds, serving as a critical analytical tool in diverse fields including food science, environmental monitoring, pharmaceutical research, and forensic analysis [23]. In this study, GC-MS employing a untargeted analytical approach comprehensively characterized volatile components, enabling systematic screening of key odorants critically contributing to strawberry aroma profiles [24]. The core advantage of GC-MS untargeted profiling lies in comprehensive coverage. Full-scan mode enables unbiased volatile organic compound (VOC) detection across complex matrices, while spectral library matching with dual-column RI alignment facilitates compound identification. This methodology exhibits methodological universality, and delivers critical datasets for downstream analyses [25]. The methodology also elucidated the impacts of postharvest treatments [26] and storage conditions [27] on flavor compound dynamics.

In this study, strawberries were treated with MAP, 1-MCP, and their combinations, and were cold stored and sampled during cold storage. Quality indicators, related enzyme activities were measured, volatile compounds detected by SPME—GC—MS with relative odor activity value (ROAV) calculation, and sensory evaluation was conducted. This study aim to reveal the influence of MAP and 1-MAP on the cold storage quality of strawberry, and investigate the flavor evolution during cold storage in detail. The results provided can add new information on the research of strawberry preservation and flavor profile change.

## 2. Materials and Methods

### 2.1. Plant Materials and Treatments

Strawberry (*Fragaria x ananassa* Duch. cv. Yuexin) were harvested from a local farm in Yangzhou, China. Fruits were in turning stage, and the color cover were around 70%. Fruit without injury and with uniform appearance were selected for treatment. To select the optimum MAP condition, based on the method proposed by Méndez-Galarraga with modifications [28], strawberry fruits were packed with atmosphere condition 5% O_2_ + 15% CO_2_ + 80% N_2_ (MAP1), 10% O_2_ + 10% CO_2_ + 80% N_2_ (MAP2), 15% O_2_ + 5% CO_2_ + 80% N_2_ (MAP3) in low density polyethylene MAP bag (thickness 40 μm, O_2_ permeability 9.2 × 10^3^ mL/(m^2^ · d), CO_2_ permeability 4.37 × 10^4^ mL/(m^2^ · d) at 20 °C and 1 standard atmosphere pressure), the fruit that not packed were set as control (CK). Note that during cold storage and simulated shelf-life, dynamic changes in headspace O_2_ and CO_2_ concentrations within the MAP packages were not continuously monitored. This experimental design focused on comparing the effects of initial gas compositions and combined treatments (1-MCP + MAP) on strawberry quality and flavor, with the expectation that the LDPE film permeability would regulate headspace gases toward a stable equilibrium (hypoxic and high CO_2_) over time, based on typical respiratory characteristics of strawberry fruit. However, the actual headspace gas profiles during storage remain unquantified in this study. To improve the storage quality of strawberry under MAP, after the selection of MAP condition, the 1-MCP treatment was added before package. Based on the method described by Chaiprasart with appropriate modifications [29], strawberry fruits were sealed in closed container (5 L) with 250 nL/L 1-MCP for 18 h at 20 °C (1-MCP), then part of fruit were packed in MAP condition (1-MCP + MAP). Following treatment and packaging, all fruit samples were stored at 4 °C with a relative humidity of 85% for 15 d. Subsequently, they were transferred to an environment of 20 °C and 80% relative humidity for 2 d to mimic the shelf—life conditions. Fruit samples were collected at 3 d intervals during cold storage and after the simulated shelf—life period. Each treatment and testing procedure was replicated three times to ensure statistical reliability.

### 2.2. Measurement of Weight Loss Rate

Based on the method described by Caleb with appropriate modifications [30], the weight of 20 strawberry fruits of each groups before storage (W_0_) and after storage (W_t_) were recorded, the weight loss rate (%) = (W_0_ − W_t_) × 100%/W_0_.

### 2.3. Measurement of Electrolyte Leakage

The determination of electrolyte leakage was conducted based on the method proposed by Kirnak with certain modifications [31]. Twenty circular strawberry fruit flesh samples, each with a thickness of 5 mm and a diameter of 5 mm, were submerged in 20 mL of distilled water for 30 min. The electrical conductivity of the 20 mL water was measured twice by a DDS—11A conductivity meter (Shanghai, China). First, prior to boiling (denoted as L0), and then after boiling the solution for 5 min, while maintaining a constant sample volume throughout the measurements (denoted as L1). The electrolyte leakage (%) = L0 × 100%/L1.

### 2.4. Measurement of Titratable Acid (TA) and Soluble Solids Content (SSC)

The determination of TA and SSC was performed with reference to the method described by Chaiprasart [29]. Total 1/4th of each group of repeated samples juiced together and used for TA and SSC measurements. For TA, mixed juice was diluted for 10 times and 50 mL of which was titrated with 0.1 N NaOH until the pH was 8.1 and expressed as % citric acid. A hand-held refractometer was used for the measurement of SSC. Three measurements were made for each test.

### 2.5. Measurement of Alcohol Dehydrogenase (ADH), Alcohol Acyl—Transferase (AAT) and Lipoxygenase (LOX) Activity

The activities ofADH, AAT, and LOX were all determined using the method described by Powers [32]. For ADH activity determination, 1 g of strawberry tissue was homogenized with 5 mL of 0.2 M Tris-HCl buffer (pH 8.0) containing 0.02 M KCl and 0.02 M EDTA. After homogenization, the solution was centrifuged at 8000× *g* for 20 min at 4 °C, the supernatant was carefully extracted. For the enzymatic assay, 0.1 mL of the supernatant was mixed with the reaction substrate, which consisted of 2.4 mL of 0.1 M Gly-NaOH buffer (pH 9.0), 100 μL of 5 mg/mL NAD, and 100 μL of ethanol. The reaction was allowed to proceed at room temperature. ADH activity was measured and expressed as U g^−1^ FW, where one unit (U) represents a 0.001 change in absorbance at 340 nm per minute.

For AAT activity determination, fresh tissue (1 g) was homogenized in 3 mL of ice-cold extraction buffer (contained 0.5 M Tris-HCl, pH 8.0, 0.1% Triton X-100, 0.3 mg PVPP), followed by centrifugation at 8000× *g* (4 °C, 20 min) to obtain a crude enzyme supernatant. The reaction mixture (2.5 mL total volume) comprised 5 mM MgCl_2_, 150 μL acetyl-CoA (5 mM in Tris-HCl buffer), and 50 μL butanol (200 mM in Tris-HCl buffer). After adding 150 μL enzyme extract, the mixture was incubated at 35 °C for 15 min, followed by 100 μL DTNB (10 mM) addition and a 10-min reaction at room temperature. AAT activity (U g^−1^ FW) was calculated based on absorbance at 412 nm, where 1 U corresponds to a 0.01 absorbance increase per minute.

For LOX activity determination, fruit tissue (1 g) was homogenized in 5 mL of 100 mM phosphate buffer (pH 8.0) supplemented with 2% PVP (*w*/*v*), centrifuged at 12,000× *g* (4 °C, 20 min), and the supernatant was subsequently collected. Clarified supernatant (0.5 mL) was combined with a reaction system consisting of 2.4 mL 100 mM phosphate buffer (pH 6.8) and 0.1 mL 10 mM sodium linoleate substrate. Enzymatic activity (U g^−1^ FW) was calculated based on absorbance at 234 nm, with 1 U corresponding to a ΔA_234_ increment of 0.1 per minute under spectrophotometric monitoring conditions.

### 2.6. Measurement of Volatile Flavor Compounds

Referring to Wang’s method with slight modifications [33]. Total 5 g samples were ground and homogenized with 1 mL saturated NaCl solution (NaCl was added to improve the adsorption capacity of the extraction head). 5 g subsamples were placed into a 20 mL glass vial and the vials were sealed tightly. The solid-phase microextraction (SPME) was performed using a 65 μm PDMS/DVB fiber (Supelco, Bellefonte, PA, USA). The fibers were placed in the headspace of the vial and incubated at 40 °C for 40 min. Subsequently, the fibers were removed and inserted into the heated injection port of a gas chromatograph (GC) for desorption at 250 °C for 5 min. For accurate quantification, 10 μL of a 1,2-dichlorobenzene solution (0.057 g/L) was added as an internal standard prior to analysis.

Volatile compound analysis utilized a dual-column GC-MS configuration (Thermo Scientific™ Trace DSQII, Thermo Fisher Scientific Inc., Waltham, MA, USA) with complementary stationary phases: polar TG-Wax and non-polar DB-5MS columns (both 30 m × 0.25 mm × 0.25 μm). The optimized temperature program initiated at 40 °C with sequential ramps: 5 °C/min → 65 °C (3 min hold), 5 °C/min → 150 °C (4 min hold), and 10 °C/min → 210 °C (2 min hold). Carrier gas (He) flow was maintained at 0.8 mL/min in constant flow mode. Injection parameters included 250 °C split injection (10:1) with EI-MS detection (70 eV) under the following conditions: ion source 150 °C, transfer line 250 °C, and mass range m/z 33–300.

The concentration of volatile compounds was semi-quantitatively estimated using the internal standard method. 10 μL of 1,2-dichlorobenzene solution (0.057 g/L) was added as the internal standard (final concentration 0.114 mg/kg). The concentration of target compounds was calculated using the formula:

$$C_{t}\;(\mu g\cdot {\mathrm{kg}}^{-1})\;=\;(A_{t}/A_{\mathrm{is}})\;\times \;(m_{\mathrm{is}}\;(\mu g)/m_{s}\;(\mathrm{kg}))$$

where: *Cₜ* = concentration of the target compound; *Aₜ* = peak area of the target compound; *Aᵢₛ* = peak area of the internal standard (1,2-dichlorobenzene); *mᵢₛ* = mass of the internal standard; *mₛ* = mass of the sample.

Due to the lack of all standard compounds, class-specific response factor correction was applied (esters: RF = 1.0; aldehydes: RF = 0.85; alcohols: RF = 0.75; terpenes: RF = 1.1; others: RF = 1.0) [17].

NOTE: This experiment used C_8_–C_20_ n-alkane standards (Sigma-Aldrich, St. Louis, MO, USA) for dual-column RI calibration. Due to limited adsorption of 65 μm PDMS/DVB SPME fibers for <C_7_ (C_3_–C_7_) volatile compounds (e.g., Ethanol, Acetaldehyde), their capture efficiency was significantly reduced, so systematic analysis of C_3_–C_7_ compounds was not performed. This limitation arises from the weak affinity of PDMS/DVB fibers for low-molecular-weight compounds, consistent with typical SPME technology characteristics.

### 2.7. Compound Identification

Preliminary identification of volatile compounds is achieved through untargeted volatile analysis based on GC-MS for full-scan detection of the analytes, followed by comparison with standard spectra retrieved from the NIST14 and Wiley 11 libraries. The definitive identification of volatile compounds was achieved by cross-referencing their retention indices (RIs) against established standards using dual-column chromatography (DB-5MS and TG-WaxMS phases). During the identification of volatile compounds in this study, the Retention Index (RI) was calculated using the Linear Retention Index (LRI) method, with the specific formula as follows:

$$LRI=100[n+(Rt(i)-Rt\left(n\right))÷\left(Rt(n+1\right)-Rt(n))]$$

where *n* and *n* + 1 represent the carbon numbers of adjacent n-alkanes flanking the compound, with corresponding retention times *Rt*(*n*) and *Rt*(*n +* 1), respectively, and *Rt*(*i*) denotes the retention time of the target analyte (*Rt*(*n*) < *Rt*(*i*) < *Rt*(*n + 1*)).

### 2.8. The Calculation of ROAV

The relative contribution of volatile compounds was assessed via the ROAV [34], computed through the formula below:

$${ROAV}_{i}=100\times C_{i}/{OT}_{i}\times T_{max}/C_{max}$$

In the equation, *C_i_* is the calculated concentration of the volatile compound in sample i, *OT_i_* is the compound’s odor threshold reported in the flavornet databasefor the aqueous phase, and max referred to compounds which have the highest odor activity value. Compounds with ROAVs greater than or equal to 1 contribute greatly to the overall flavor.

### 2.9. Descriptive Sensory Analysis

Descriptive sensory analysis was performed by trained sensory evaluators comprising five males and five females aged between 22 and 45 years, all from Yangzhou University. Panelists requested general training was carried out four two-hour sessions within two weeks. During this period, the panelists evaluated the sample respectively, and then wrote down the descriptors that could represent the characteristics of the sample. Then, the team leader discussed the collected descriptors several times to achieve agreement. Finally, five kinds of aroma attributes are used. Samples (5.0 g) were aliquoted into 15 mL polypropylene sensory cups labeled with randomized codes. Under standardized conditions (25 °C), panelists quantified aroma attributes using a 15-point intensity scale, with triplicate evaluations separated by 1-min inter-sample intervals to prevent olfactory adaptation [35]. The sensory evaluation experiment was performed in complicance with the laws of the People’s Republic of China and institutional guidelines of Yangzhou University. The experiment also was approved by the Ethics Committee of Yangzhou University. All volunteers were mentally and physically able to participate in the sensory evaluation and an informed written consent was obtained from each volunteer.

### 2.10. Statistical Analysis

The experimental design followed a complete randomized protocol. Appropriate statistical analysis methods were selected based on the type of measurement: for non-destructive measurement indicators such as weight loss rate, the data were analyzed using repeated-measures analysis of variance (repeated-measures ANOVA); for destructive measurement indicators such as titratable acidity (TA) and electrolyte leakage, the data were analyzed using two-way analysis of variance (Two-way ANOVA), with the interaction effect between treatment method and storage time included in the analysis to capture dynamic differences. All quantitative results were expressed as “mean ± standard deviation (SD) (*n* = 3)” and statistically validated via Tukey’s post-hoc test in SPSS 25.0, with the significance level set at *p* < 0.05. Multivariate analysis incorporated chemometric PLSR modeling of aroma profiles via the Unscrambler X 10.4, complemented by hierarchical clustering heatmaps generated using R v4.1.2 (pheatmap 1.0.8).

## 3. Results

### 3.1. Effects of MAP on the Cold Storage Quality of Strawberry

**Figure 1:**
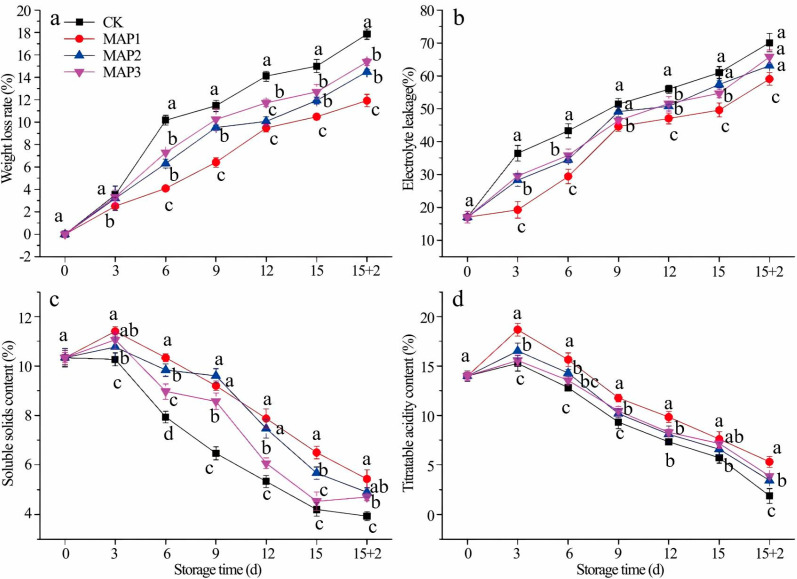
Effects of MAP on weight loss rate (**a**), electrolyte leakage (**b**), soluble solids contents (**c**), titratable acidity contents (**d**) of strawberry under cold storage. Refrigeration conditions: stored at 4 °C with a relative humidity (RH) of 85%. Shelf-life conditions: placed at 20 °C with a relative humidity (RH) of 80% for 2 days. Different letters indicate significant differences within the same storage day (*p* < 0.05).

Figure 1 delineates the storage dynamics of MAP-packaged strawberries, tracking: (a) weight loss rate, (b) electrolyte leakage, (c) soluble solids content (SSC), and (d) titratable acidity (TA). Progressive storage duration exhibited significant increases in mass loss and membrane permeability, contrasted by SSC and TA depletion. MAP1 implementation effectively mitigated physiological deterioration, suppressed weight loss and electrolyte leakage escalation by 38% and 45% respectively, while maintained 28% higher SSC and 33% greater TA retention compared to controls. MAP1 condition exhibited most significant inhibitory effect of 4 indexes, MAP2 and MAP3 showed no obvious difference, so MAP1 were chosen for the best MAP condition.

### 3.2. Effects of 1-MCP and MAP on the Cold Storage Quality of Strawberry

**Figure 2:**
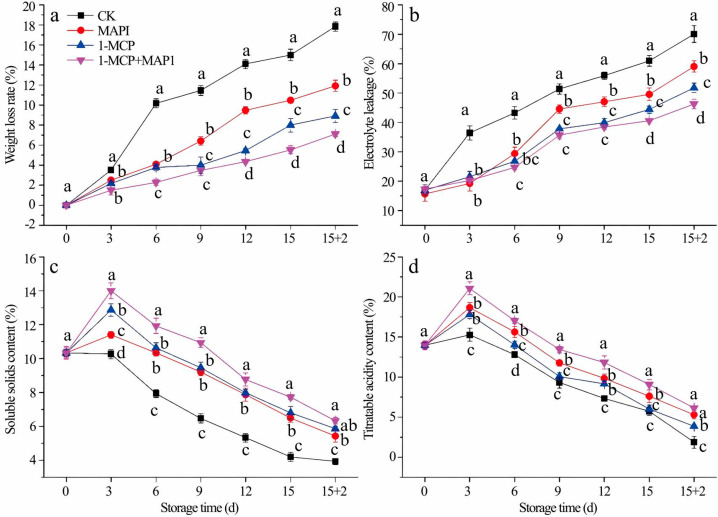
Effects of MAP and 1-MCP on weight loss rate (**a**), electrolyte leakage (**b**), soluble solids content (**c**), titratable acidity content (**d**) of strawberry under cold storage. Refrigeration conditions: stored at 4 °C with a relative humidity (RH) of 85%. Shelf-life conditions: placed at 20 °C with a relative humidity (RH) of 80% for 2 days. Different letters indicate significant differences within the same storage day (*p* < 0.05).

Building upon optimized MAP parameters, 1-MCP treatment was integrated to enhance strawberry postharvest quality. As detailed in Figure 2, combined effects were observed across key physiological metrics: (a) mass loss rate, (b) membrane permeability, (c) soluble solids content (SSC), and (d) titratable acidity (TA). Comparative analysis revealed 1-MCP’s superior efficacy, achieving 53.6 ± 2.1% (*p* < 0.01) reduction in mass loss and 59.4 ± 3.0% (*p* < 0.01) suppression of membrane permeability versus MAP alone, though SSC (Δ = 1.2 ± 0.5%) and TA (Δ = 1.8 ± 0.6%) showed non-significant variation (*p* > 0.05). The combined 1-MCP-MAP1 protocol demonstrated optimal performance, limited mass loss to 15.3% (vs. 42.1% in controls) and membrane permeability to 18.7% (vs. 58.4% in controls), while preserved SSC at 14.5 ± 0.3°Brix and TA at 0.89 ± 0.04%, represented 31% and 37% retention advantages over conventional storage.

### 3.3. Effects of 1-MCP and MAP on the ADH, AAT, LOX Activities of Strawberry Under Cold Storage

**Figure 3:**
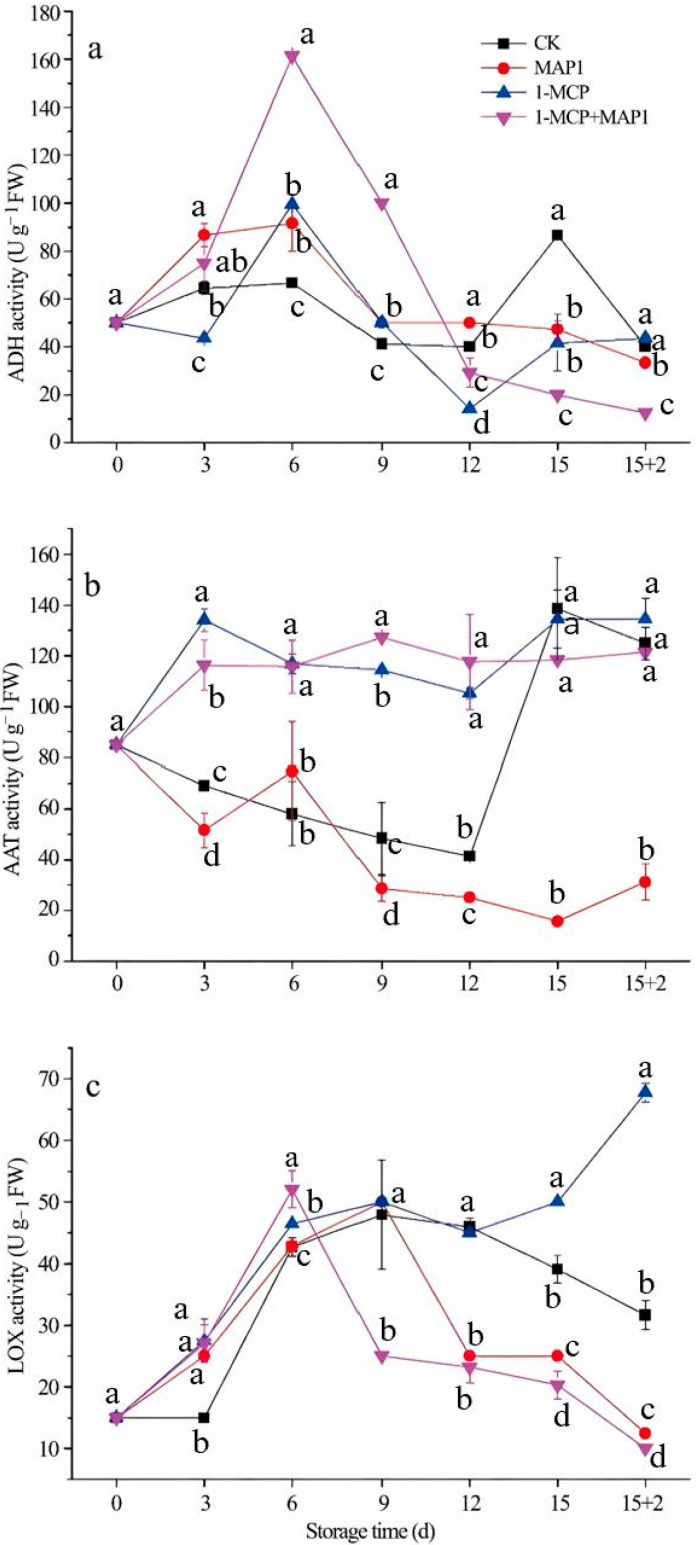
Effects of 1-MCP and MAP on the ADH (**a**), AAT (**b**), LOX (**c**) activities of strawberry under cold storage. Refrigeration conditions: stored at 4 °C with a relative humidity (RH) of 85%. Shelf-life conditions: placed at 20 °C with a relative humidity (RH) of 80% for 2 days. Different letters indicate significant differences within the same storage day (*p* < 0.05).

ADH activity across treatment groups peaked at day 6 of cold storage, followed by progressive attenuation throughout the remaining storage period (Figure 3a). Notably, CK and 1-MCP-treated fruits displayed a resurgence of enzymatic activity during terminal storage phases (days 12–15). Fruits treated with 1-MCP alone or in combination with MAP1 displayed the highest ADH activity at day 6 but the lowest activity by the storage endpoint. Conversely, CK fruits showed the lowest ADH activity at day 6 but the highest activity at storage termination. For AAT activity, CK and MAP1-treated fruits showed a general decline during cold storage, with the exception of a sharp increase observed in CK fruits at the storage endpoint (Figure 3b). In contrast, 1-MCP-treated and 1-MCP + MAP1-treated fruits exhibited increased AAT activity starting at day 3, maintained high levels throughout the storage period. LOX activity in all fruit groups peaked at day 9, except for 1-MCP + MAP1-treated fruits peaked at day 6 (Figure 3c). 1-MCP-treated fruits displayed elevated LOX activity during late storage, while MAP1-treated and 1-MCP + MAP1-treated fruits maintained lower LOX activity levels.

### 3.4. Effects of 1-MCP and MAP on the Volatile Flavor Compounds of Strawberry Under Cold Storage

Table 1 presents the volatile profiles of strawberry after sequential storage phases: 15 days under refrigerated conditions (4 °C) and followed by 2-day shelf-life simulation (20 °C). A total of 85 VOCs were detected, comprising 34 esters, 17 aldehydes, 13 hydrocarbons, 4 alcohols, 3 acids, 3 ketones, 3 furans, 4 terpenoids, 1 lactone, and 3 other compounds. Among the nine tested samples, ester and aldehyde compounds constituted the primary volatile components, accounting for 76–98% of the total volatile content (Table 2). Specifically, acetates were the most abundant class with 10 identified compounds, followed by butanoates and hexanoates. Among the nine samples, 2-hexenol acetate (green fruity flavor) was most abundant, reached 389.25 μg/kg after 15 days of MAP1 treatment—2.02× higher than CK (Table 3). Low-threshold compounds including ethyl hexanoate, hexyl acetate, and amyl acetate were identified, being easily detectable by consumers and critical for fruit flavor/quality assessment. Alcohols and furans were confirmed as major strawberry volatiles, with their composition and concentration significantly influenced by treatments and storage duration.

ROAV serves as a critical metric for assessing individual compounds’ contributions to strawberry aroma profiles. Compounds with ROAV ≥1 were designated as key aroma-active constituents (Table 4), including:

Esters: Ethyl hexanoate (E1), (Z)-3-Hexenyl acetate (E4), Hexyl acetate (E5), Benzyl acetate (E9), Ethyl octanoate (E12), Ethyl butanoate (E23), Ethyl 2-methylbutanoate (E26), Banana oil (E27), Amyl acetate (E29).

Aldehydes: (E)-2-Octenal (A1), Hexanal (A5), Nonanal (A6), *trans*-2-*trans*-6-Nonadienal (A7), 2-Nonenal (A8), (E,E)-2,4-Nonadienal (A10), 2,4-Decadienal (A12), Undecanal (A13), Dodecanal (A15).

Ketones/Terpenes/Lactones: 1-Octen-3-one (K1), DMMF (F1), DMHF (F2), Linalool (T2), γ-Dodecalactone (L1).

This classification system, based on ROAV calculations integrated quantitative GC-MS data and literature-derived odor thresholds, highlighted these compounds as primary drivers of strawberry’s characteristic aroma profile under postharvest conditions.

Esters represented the largest category in the nine groups of different samples accounting for 17–88% of all strawberry volatiles (Table 2). On the 15th day, the total ester content in 1-MCP + MAP1 treatment had no difference with 1-MCP treatment, but both lower than CK and MAP1 treatment, and esters in MAP1 was the highest. The total content of volatiles esters reached the highest during cold storage, and then decreased rapidly during the shelf-life period, except for 1-MCP treatment. Notably, the benzyl acetate concentration in the 1-MCP + MAP1 treated fruit after shelf life substantially exceeded that of both the control group and other treatments (Table 3), enhancing the fruit’s sweet, floral, and fresh aromatic profile. While ethyl hexanoate, ethyl butanoate, and ethyl octanoate levels generally rose from cold storage to shelf life across most treatments, MAP1 alone showed an inverse trend—these esters peaked post-cold storage before declining. Similarly, hexyl acetate and amyl acetate accumulated during refrigeration but diminished quickly once the fruit reached shelf conditions. Together, these volatile compounds played a key role in defining the fruit’s distinctive fragrance.

Aldehydes represent a major class of volatile compounds that significantly influence strawberry flavor profiles. Hexanal, in particular, imparts characteristic green, grassy notes. This investigation revealed a marked reduction in hexanal levels among treated samples when compared to the control (CK) group during both refrigerated storage and subsequent shelf life evaluation (Table 3). At the conclusion of cold storage, the 1-MCP + MAP1 treatment exhibited the lowest hexanal concentration, though its hexanal levels surpassed those of the MAP1-only treatment during shelf life. Regarding aldehyde trends overall, concentrations reached their maximum at the onset of cold storage (day 0) before steadily declining over time. During post-refrigeration shelf life, all treatments exhibited a continued decrease in aldehyde content—except for 1-MCP + MAP1, which showed divergent behavior.

Alcohol acts as a key building block for ester formation. Although no traces of alcohols were found in the samples throughout cold storage, these compounds emerged during the shelf-life phase (Table 3). Notably, fruits treated with 1-MCP + MAP1 showed substantially greater alcohol accumulation than those subjected to alternative preservation methods.

When examining furan compounds, the treatment combining 1-MCP and MAP1 maintained minimal levels until the conclusion of cold storage but rebounded during shelf life (Table 3). Notably, 2,5-Dimethyl-4-methoxy-3(2H)-furanone (DMMF) saw a substantial rise in both the 1-MCP and MAP-treated samples, peaked particularly in the CK15 + 2 and 1-MCP 15 + 2 groups. This suggests that these treatments could enhance DMMF production, likely tied to oxidative processes or shifts in strawberry metabolism during storage. Meanwhile, 2,5-Dimethyl-4-hydroxy-3(2H)-furanone (DMHF) remained barely detectable, appearing only in the CK15 + 2 and 1-MCP 15 + 2 groups. This suggests that the generation of DMHF is influenced by the storage time and treatment methods of strawberries, but its impact on the overall flavor may be limited. The content of trans-Linalool oxide (furanoid) was the highest in the 1-MCP 15 group, indicating that the 1-MCP treatment may promote its generation. This compound, typically associates with floral and fruity aromas, may positively contribute to strawberry aroma. In groups like CK15 + 2, furan compound content increased significantly with extended storage time, potentially linked to oxidation and metabolic reactions during storage. The 1-MCP treatment may delay strawberry ripening and senescence by inhibiting ethylene action, thereby influence furan compound generation. The application of MAP alone showed limited influence on furan compound levels, yet its combined use with 1-MCP resulted in significant accumulation of these compounds. This combined effect suggests that MAP might facilitate furan formation through secondary regulation of storage atmosphere composition, particularly O_2_ and CO_2_ concentrations.

### 3.5. Descriptive Sensory Analysis

The acidity scores of different treatment groups showed significant differences (Table 5), the CK0 group had the highest acidity score (8.67 ± 0.58 a), while the CK15 + 2 group had the lowest (5.67 ± 0.58 b). This indicates that 1-MCP and MAP treatments may have a certain impact on the acidity of strawberries. The sweetness evaluation revealed marked variations across experimental groups, with the control (CK15) exhibited the highest scores (7.67 ± 0.58 a) and the 1-MCP 15 + 2 recorded the lowest values (5.33 ± 1.53 ab) (Table 5). This differential pattern suggests that 1-MCP application could potentially inhibit sweetness development in strawberry fruits. The 1-MCP + MAP1 15 group had the highest ripe fruit smell score (7.33 ± 1.15 a), while the CK0 group had the lowest score (6 ± 1 b). While aroma persistence scores showed no significant variation across treatment groups, the highest value (9 ± 1 a) was recorded in the 1-MCP 15 + 2 group, indicating a potential role of 1-MCP in enhancing aroma retention in strawberries. Significant variations in alcoholic odor scores were observed across treatments, with the 1-MCP 15 + 2 group exhibited the highest value (9 ± 1 a) and the CK0 group showed the lowest (4 ± 0 b). These results suggest that although 1-MCP application enhances strawberry shelf life, it may also promote the development of alcoholic off-flavors. The 1-MCP + MAP1 15 group had the highest acceptability score (8.33 ± 1.15 a), while the MAP1 15 + 2 group had the lowest score (6.67 ± 1.15 a) (Table 5).

1-MCP and MAP treatments significantly influence the sensory attributes of strawberries, particularly in preserving or enhancing ripe fruit aroma and overall acceptability. However, 1-MCP treatment may lead to a reduction in sweetness and an increase in alcoholic smell, which need to be balanced in preservation treatments.

### 3.6. PLSR Analysis

**Figure 4:**
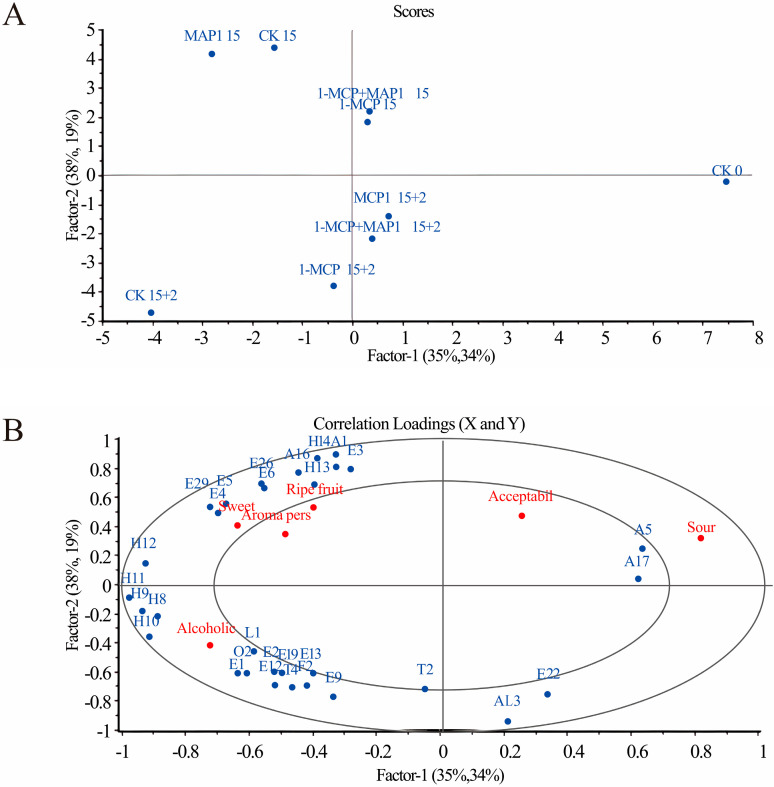
The volatile compounds isolated from GC-MS as X-matrix and sensory properties as Y-matrix, the PLSR models of nine groups of strawberry samples were established. (**A**) PLSR Scores Plot, (**B**) PLSR Correlation Loading Plot. The code is from Table 3. Refrigeration conditions: stored at 4 °C with a relative humidity (RH) of 85%. Shelf-life conditions: placed at 20 °C with a relative humidity (RH) of 80% for 2 days.

PLSR models were developed using GC-MS-derived volatile compounds (X-matrix) and sensory evaluations (Y-matrix) across nine strawberry sample groups. The PLSR scores plot (Figure 4A) revealed distinct clustering of treatments along principal components 1 and 2, reflecting separation based on aroma profiles and sensory attributes. The correlation loading plot (Figure 4B) demonstrated that 73% of instrumental variance explained 53% of sensory variance, highlighting the interplay between volatile composition and perceived quality. Sour, sweet and alcoholic smell are located between the inner and outer oval, and were significantly related to aromatic compounds. Among them, sweet is related to (Z)-3-Hexenyl acetate (E4), Hexyl acetate (E5), Amyl acetate (E29), while alcoholic may be jointly determined by Ethyl hexanoate (E1), Ethyl benzoate (E2), Ethyl octanoate (E12), Ethyl caprinate (E19), Triethyl phosphate (O2) and γ-Dodecalactone (L1).

### 3.7. Systematic Clustering and Heat Map Analysis

**Figure 5:**
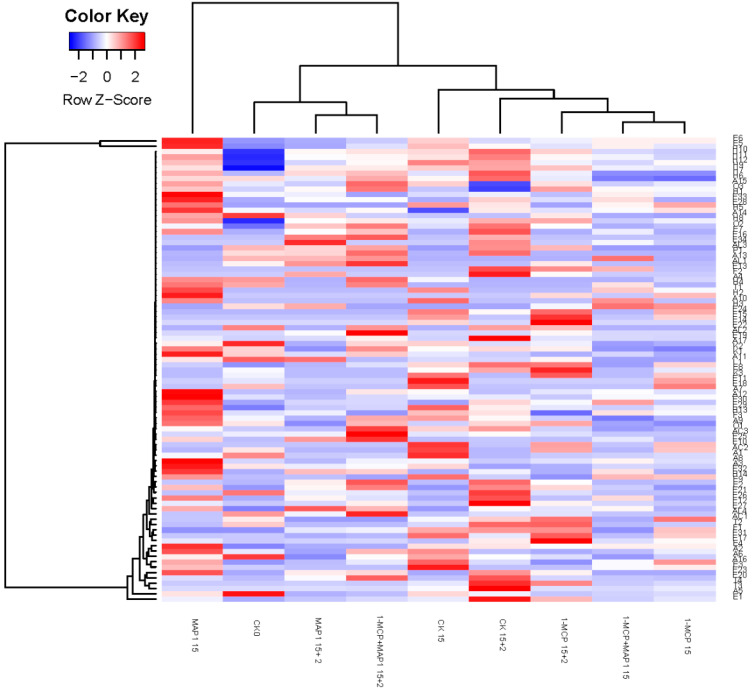
Hierarchical clustering and heat map visualization of the concentration of volatile compounds. Blue, white and red indicate that the concentration of volatile compounds ranges from low to high. Volatile compounds are represented by code as shown in Table 3. Nine groups of treatments were clustered, which were CK0, CK15, 1-MCP15, MAP1 15, 1-MCP + MAP1 15, CK15 + 2, 1-MCP15 + 2, MAP1 15 + 2, 1-MCP + MAP1 15 + 2. Refrigeration conditions: stored at 4 °C with a relative humidity (RH) of 85%. Shelf-life conditions: placed at 20 °C with a relative humidity (RH) of 80% for 2 days.

Systematic clustering and heat map analysis revealed the diversity and concentration of volatile aroma compounds (Figure 5). Here, the *Y*-axis represents the “Euclidean distance” between volatile compounds, quantifying the similarity of their concentration change trends across all treatment groups. A smaller distance indicates more similar trends—e.g., ester compounds hexyl acetate (E5) and 2-hexenol acetate (E6), which both accumulate during cold storage and decrease during shelf life, cluster closely. Conversely, a larger distance reflects more distinct trends, such as aldehyde hexanal (A5, decreasing with storage) and furan DMMF (F1, rising in shelf life), which lie in separate branches. Note that this distance does not reflect concentration (indicated by heat map colors: blue for low, red for high) but metabolic relevance—e.g., esters like E1, E5, and E29 cluster closely, suggesting regulation by similar mechanisms (e.g., AAT enzyme dependence). Clustering results show MAP1 15 + 2 and 1-MCP + MAP1 15 + 2 group with CK0, while other samples (except MAP1 15) form a separate cluster. This indicates the former two treatments are similar to CK0 in volatile types and quantities, confirming they help most volatiles (especially key esters) maintain trends closest to fresh samples, thus retaining fresh-like volatile characteristics.

## 4. Discussion

Strawberry, a non-climacteric fruit, undergoes sequential physiological and molecular events during development and ripening, inducing marked transformations in size, pigmentation, texture, taste, and volatile profiles, with a spectrum of postharvest losses occurring post-harves [36]. 1-MCP, an ethylene biosynthesis inhibitor, is widely applied in postharvest management of climacteric fruits due to its capacity to suppress ethylene-mediated ripening processes [37]. It is widely applied to fruits such as apples [38], pears [39], and persimmons [40]. In addition, it is also used for non-climacteric fruits [41,42,43,44]. Evidence indicates that low-dose 1-MCP application effectively retards postharvest strawberry ripening while preserving fruit physiological integrity [45]. Non-climacteric fruits such as strawberries exhibit low ethylene production and mild ethylene-mediated effects. Low-dose 1-MCP can competitively bind to the limited ethylene receptors, thereby blocking ethylene signaling. This not only effectively inhibits weight loss and electrolyte leakage to delay ripening but also avoids excessive interference with normal fruit metabolism. Such a mechanism is well-suited to the characteristics of non-climacteric fruits and represents a key factor for efficient preservation. In our study, a similar conclusion was drawn, 1-MCP could effectively inhibit the weight loss rate and electrolyte leakage rate [41]. The electrolyte leakage rate directly reflects the degree of cell membrane damage [31]. The collapse of the membrane system leads to the leakage of nutrients, accelerating the infection of pathogens such as Botrytis cinerea [3,46]. In this study, 1-MCP + MAP1 reduced the leakage rate, explaining the mechanism behind the decrease in decay rate. This study did not directly quantify the decay rate, but cross validation was conducted through the electrolyte leakage rate and the value of sensory acceptance. Future research needs to add the measurement of decay spot diameter to improve this indicator. Ethylene binds to fruit ethylene receptors, activating signaling pathways to regulate membrane permeability, metabolism, and senescence-related gene expression, accelerating softening, weight loss, and flavor deterioration. 1-MCP blocks this signal via competitive receptor binding, inhibiting such effects [44,47]. Some studies indicate that the quality indicators such as hardness and weight loss rate of fruits treated with MAP are significantly improved. It is believed that low oxygen inhibits fruit metabolism, and high carbon dioxide inhibits the production of ethylene [42]. Some literatures have used 1-MCP combined with CaCl_2_ [48] or MAP [44] composite packaging materials. The combined application of MAP with calcium chloride treatment enhances 1-MCP-mediated ethylene suppression efficacy [49]. In this study, the combined 1-MCP and MAP1 treatment demonstrated combined efficacy, resulting in superior strawberry quality compared to both control and alternative experimental groups.

In order to explore the change mechanism of flavor substances during the interaction between 1-MCP and MAP in the postharvest storage of strawberries, this study introduced untargeted analytical method. Untargeted analysis employs high-resolution gas chromatography-mass spectrometry (HRGC-MS) in full-scan mode to comprehensively analyze volatile compounds in strawberry samples [50]. Untargeted analysis detected 85 volatile metabolites in strawberries across four experimental groups, encompassing diverse chemical classes including esters, aldehydes, alcohols, and terpenoids. These compounds form the unique flavor profile of strawberries, this result is consistent with the general research characteristics of strawberry VOCs: a review by Yan et al. pointed out that more than 360 VOCs have been identified in strawberries, but only a few actually contribute to the flavor, and esters, aldehydes, and furans are the core categories. Untargeted volatile profiling data served as the foundation for calculating ROAVs, enabling systematic prioritization of key flavor contributors exhibiting ROAV ≥ 1. This threshold-based prioritization facilitates unambiguous discrimination of sensorially decisive compounds among the 85 detected volatiles, thereby establishes a focused analytical paradigm for evaluating treatment-specific effects on strawberry aroma profiles. Research shows that the formation of volatile compounds in strawberries depends to a large extent on the storage process [51]. Untargeted analysis of data indicates that with prolonged storage, ester content in control strawberries declines rapidly, while aldehyde and alcohol levels increase significantly, which is closely associated with fruit senescence. Although the 1-MCP treatment delays fruit aging, analysis of key aroma compounds reveals that synthesis of characteristic ester aroma compounds is inhibited, leading to reduced perception of sweetness and increased alcohol-related flavor substances.

Judged from the results of sensory evaluation and instrumental tests, the 1-MCP + MAP1 group scored high in “ripe fruit smell,” which was associated with the retention of relatively high levels of esters such as hexyl acetate (E5) and 2-hexenol acetate (E6) (Table 3); PLSR analysis confirmed that these esters (with ROAV ≥ 1) were positively correlated with this attribute (Figure 4), this is consistent with the conclusion of Fan et al. that “strawberry sweetness and consumer preference are positively correlated with the concentration of specific esters” [15], and the combined treatment promoted ester synthesis by maintaining AAT enzyme activity (Figure 3b),This is consistent with existing studies on metabolic pathways: AAT catalyzes the combination of alcohols and acyl-CoA to form esters [14]. The 1-MCP alone treatment had a prominent “alcoholic smell,” which was related to the accumulation of (Z)-2-hexen-1-ol (AL4) and DMMF (F1) during the shelf-life period (Table 3), as it failed to effectively regulate ADH-mediated alcohol synthesis (Figure 3a), while the combined effect of MAP could alleviate this problem by optimizing gas composition. The high acidity score of the CK0 group was associated with its titratable acid content (Figure 1d and Figure 2d), and the 1-MCP + MAP1 treatment moderately retained acidity (6.33 ± 1.53), balancing the sweet-sour ratio. The high acceptability of the 1-MCP + MAP1 group stemmed from the enhancement of “ripe fruit smell” and the inhibition of “alcoholic smell,” which was consistent with the heatmap showing that its volatile components were close to those of CK0 (Figure 5), indicating that sensory quality depends on the balance between characteristic aromas and off-flavors. In previous studies, similar results have proven that 1-MCP treatment is likely to inhibit the development of aroma [52,53] and affect the flavor of fruits [54].

Untargeted analysis in this study further reveals the combined mechanism: 1-MCP slows down the physiological aging process of fruits by inhibiting ethylene action, which provides a basis for the stable synthesis of flavor substances; MAP inhibits the excessive metabolism of fruits by regulating the gas environment and reduces off-flavor compound production. When the two methods are used in combination, they show the best effect in maintaining the acidity, aroma, and acceptance of strawberries. Analysis of key aroma compounds shows that the content of characteristic aroma substances such as esters remains relatively stable during storage in the 1-MCP + MAP1 treatment group, and meanwhile the generation of off-flavor compound such as aldehydes and alcohols is significantly suppressed. Thus, it offsets the negative impacts of a decrease in sweetness and an increase in alcohol flavor caused by the single use of 1-MCP, achieving effective maintenance of the sensory quality of strawberries.

The physiological basis of combined 1-MCP and MAP action warrants further investigation, though our results demonstrate their complementary preservation effects. GC-MS-based untargeted analysis not only provided a powerful tool for analyzing flavor changes at the molecular level in this study, but also offers important research ideas and methodological support for further exploring the combined mechanism between the two and optimizing strawberry preservation technology.

## 5. Conclusions

This study demonstrates that the combined application of 1-methylcyclopropene (1-MCP; 250 nL/L for 18 h at 20 °C) and modified atmosphere packaging (MAP; 5% O_2_, 15% CO_2_, 80% N_2_) constitutes an effective strategy for delaying postharvest senescence and preserving storage quality in strawberries during cold storage and subsequent shelf life. Untargeted GC-MS analysis revealed that this combined treatment distinctly regulated volatile profiles: it significantly suppressed the formation of fatty acid oxidation-derived volatile organic compounds (VOCs), frequently associated with off-flavors, while effectively stabilizing critical aroma-active esters. Consequently, strawberries subjected to the 1-MCP + MAP treatment maintained volatile profiles most comparable to freshly harvested fruit and exhibited superior consumer acceptability. These findings underscore the potential of combined 1-MCP and MAP as a practical postharvest intervention and highlight the utility of untargeted GC-MS analysis for the scientific evaluation of flavor quality dynamics in fruit preservation technologies.

## Figures and Tables

**Table 1:** Volatile components categories of strawberry during cold storage and shelf life.

	CK 0	CK 15	MAP1 15	1-MCP 15	1-MCP + M AP1 15	CK 15 + 2	MAP1 15 + 2	1-MCP 15 + 2	1-MCP + MAP1 15 + 2
**Esters**	11	18	22	20	23	28	22	25	23
**Aldehydes**	16	16	16	15	16	12	15	12	16
**Hydrocarbons**	8	10	12	12	13	11	12	13	12
**Alcohols**	3	0	0	0	0	2	4	4	4
**Acids**	3	3	2	3	2	2	1	3	1
**Ketones**	3	3	2	3	2	2	2	3	2
**Furans**	1	2	1	2	1	3	3	3	2
**Terpenoids**	2	4	2	4	1	3	2	3	0
**Others**	2	4	4	4	4	3	4	4	4
**Total**	49	60	61	63	62	66	65	70	64

NOTE: Refrigeration conditions: stored at 4 °C with a relative humidity (RH) of 85%. Shelf-life conditions: placed at 20 °C with a relative humidity (RH) of 80% for 2 days.

**Table 2:** Volatile components contents of strawberry during cold storage and shelf life.

	CK 0	CK 15	MAP1 15	1-MCP 15	1-MCP + M AP1 15	CK 15 + 2	MAP1 15 + 2	1-MCP 15 + 2	1-MCP + MAP1 15 + 2
Concentrations (μg/kg eq.)									
**Esters**	27.7	413.56	749.2	277.8	302.24	382.82	117.57	291.54	227.57
**Aldehydes**	93.59	88.76	86.66	55.99	42.37	47.66	29.16	34.11	49.69
**Hydrocarbons**	3.2	7.37	8.68	6.12	6.42	5.45	4.37	3.35	3.82
**Alcohols**	11.01	0	0	0	0	1.75	3.54	2.39	19.37
**Acids**	9.62	9.03	3.29	17.46	1.49	11.02	0.56	18.18	2.3
**Ketones**	1.56	2.06	1.89	1.23	0.75	1.01	0.76	2.07	1.43
**Furans**	1.04	10.71	1.62	8.8	0.6	11.47	4.93	11.25	5.79
**Terpenoids**	9.74	10.03	0.35	5.13	0.02	85.7	1.02	41.33	0
**Others**	1.07	2.42	2.02	1.73	1.07	3.41	1.70	2.48	3.45
**Total**	158.53	543.94	853.71	374.26	354.96	550.29	163.61	406.7	313.42

NOTE: Refrigeration conditions: stored at 4 °C with a relative humidity (RH) of 85%. Shelf-life conditions: placed at 20 °C with a relative humidity (RH) of 80% for 2 days.

**Table 3:** Effects of 1-MCP and MAP on the volatile components of strawberry during cold storage and shelf life.

			Concentrations (μg/kg eq.)								
VOC	Code	LRI	CK 0	CK 15	MAP1 15	1-MCP 15	1-MCP + MA P1 15	CK 15 + 2	MAP1 15 + 2	1-MCP 15 + 2	1-MCP + MAP1 15 + 2
Esters											
Ethyl hexanoate	E1	1008	ND	16.45 ± 0.11 cd	17.6 ± 1.11 c	14.89 ± 0.14 cd	9.59 ± 0.82 ef	81.46 ± 6.68 a	6.79 ± 0.68 f	40.97 ± 2.78 b	12.18 ± 0.10 de
Ethyl benzoate	E2	1181	ND	ND	2.13 ± 0.18 b	ND	0.68 ± 0.01 e	2.80 ± 0.2 a	1.42 ± 0.04 d	1.81 ± 0.15 c	2.91 ± 0.08 a
Propyl myristate	E3	1872	1.42 ± 0.05 e	31.49 ± 0.29 a	14.37 ± 0.03 b	9.04 ± 0.60 c	6.46 ± 0.22 d	ND	ND	ND	ND
(Z)-3-Hexenyl acetate	E4	1012	ND	10.61 ± 0.10 b	22.23 ± 1.07 a	9.61 ± 0.47 c	9.52 ± 0.40 c	10.25 ± 0.38 bc	2.63 ± 0.20 e	8.28 ± 0.73 d	2.53 ± 0.01 e
Hexyl acetate	E5	1021	6.18 ± 0.03 f	140.51 ± 9.49 b	242.52 ± 11.4 a	72.26 ± 3.26 d	102.53 ± 2.67 c	96.22 ± 6.33 c	28.24 ± 2.27 e	70.49 ± 2.13 d	68.25 ± 2.05 d
2-Hexenol acetate	E6	1023	11.72 ± 0.23 h	193.08 ± 9.01 b	389.25 ± 16.74 a	155.27 ± 6.03 c	155.55 ± 12.91 c	88.51 ± 3.00 e	35.22 ± 1.8 g	116.55 ± 9.16 d	72.92 ± 1.57 f
Ethyl 2-hexenoate	E7	1055	ND	ND	0.57 ± 0.03 b	ND	0.18 ± 0.01 e	0.73 ± 0.01 a	0.23 ± 0.02 d	ND	0.42 ± 0.03 c
Methyl caprylate	E8	1133	0.21 ± 0.00 c	0.24 ± 0.02 c	ND	0.15 ± 0.00 d	ND	0.53 ± 0.00 b	ND	0.93 ± 0.08 a	ND
benzyl acetate	E9	1172	0.24 ± 0.01 e	0.35 ± 0.00 e	0.55 ± 0.04 e	0.41 ± 0.00 e	0.3 ± 0.01 e	4.87 ± 0.44 b	2.13 ± 0.17 c	1.81 ± 0.12 d	5.20 ± 0.01 a
Hexyl butyrate	E10	1201	ND	2.95 ± 0.18 a	ND	1.43 ± 0.01 c	ND	ND	ND	1.79 ± 0.04 b	ND
2(E)-Hexenyl butanoate	E11	1203	0.14 ± 0 c	1.91 ± 0.08 a	ND	1.01 ± 0.01 b	ND	ND	ND	ND	ND
Ethyl octanoate	E12	1206	ND	ND	1.31 ± 0.11 c	ND	0.56 ± 0.06 d	12.05 ± 0.66 a	0.62 ± 0.04 d	2.78 ± 0.15 b	2.89 ± 0.21 b
octyl acetate	E13	1221	ND	ND	ND	ND	0.20 ± 0.02 c	0.35 ± 0.01 a	ND	0.26 ± 0.02 b	ND
Hexyl isopentanoate	E14	1253	ND	0.13 ± 0.01 b	ND	ND	ND	ND	ND	0.45 ± 0.01 a	ND
trans-2-Hexenyl isovalerate	E15	1255	ND	0.24 ± 0.02 b	ND	0.16 ± 0.00 c	ND	ND	ND	0.39 ± 0.01 a	ND
β-Phenethyl acetate	E16	1265	ND	ND	ND	ND	ND	0.42 ± 0.02 c	0.56 ± 0.01 b	ND	0.66 ± 0.06 a
Methyl cinnamate	E17	1397	0.46 ± 0.04 g	ND	1.63 ± 0.08 d	2.93 ± 0.13 c	1.28 ± 0.07 e	3.57 ± 0.15 b	0.77 ± 0.04 f	12.64 ± 0.49 a	0.95 ± 0.01 f
trans-2-Hexenyl hexanoate	E18	1398	0.38 ± 0.01 c	0.69 ± 0.05 a	ND	0.56 ± 0.04 b	ND	ND	ND	ND	ND
Ethyl caprinate	E19	1405	ND	ND	0.12 ± 0.01 c	0.01 ± 0 de	0.03 ± 0 de	1.55 ± 0.05 a	0.04 ± 0 d	0.09 ± 0.01 c	0.32 ± 0 b
Ethyl cinnamate	E20	1478	ND	0.79 ± 0.03 ef	4.21 ± 0.37 d	0.64 ± 0.03 ef	1.46 ± 0.07 e	30.88 ± 1.52 a	8.16 ± 0.16 c	3.23 ± 0.14 d	29.33 ± 0.79 b
2-Ethylhexyl salicylate	E21	1836	5.13 ± 0.40 b	ND	ND	ND	ND	6.49 ± 0.07 a	1.14 ± 0.01 c	0.73 ± 0.03 b	1.01 ± 0.04 c
Dibutyl phthalate	E22	1948	0.52 ± 0.05 a	ND	ND	ND	ND	0.45 ± 0.00 c	ND	0.37 ± 0.01 d	0.48 ± 0.01 b
Ethyl butanoate	E23	807	ND	ND	27.47 ± 1.63 a	ND	ND	21.07 ± 0.18 b	11.63 ± 0.14 d	10.77 ± 0.51 d	13.55 ± 0.20 c
Isopropyl butyrate	E24	843	ND	0.28 ± 0.02 b	ND	0.22 ± 0.02 c	ND	0.11 ± 0.01 d	ND	0.31 ± 0.00 a	ND
Methacrylic acid, ethyl ester	E25	847	ND	ND	0.85 ± 0.03 c	ND	0.31 ± 0.02 d	0.07 ± 0.00 e	1.28 ± 0.04 c	ND	2.01 ± 0.01 a
Ethyl 2-methylbutanoate	E26	851	ND	ND	8.16 ± 0.32 b	ND	4.74 ± 0.34 c	9.92 ± 0.60 a	3.63 ± 0.11 d	0.79 ± 0.05 f	2.00 ± 0.02 e
Isopentyl acetate	E27	881	ND	0.79 ± 0.02 f	7.03 ± 0.16 b	1.55 ± 0.13 e	4.50 ± 0.05 c	4.38 ± 0.09 c	9.64 ± 0.49 a	3.23 ± 0.21 d	6.70 ± 0.55 b
Prenyl acetate	E28	920	ND	0.41 ± 0.03 b	0.49 ± 0.01 a	0.38 ± 0 c	0.22 ± 0.01 e	0.25 ± 0.02 d	ND	ND	ND
Amyl acetate	E29	922	ND	1.77 ± 0.03 a	1.74 ± 0.16 a	0.70 ± 0.04 c	0.50 ± 0.04 d	0.92 ± 0.02 b	0.47 ± 0.01 d	0.65 ± 0.00 c	0.54 ± 0.03 d
2-Methyl-2-butenyl acetate	E30	930	ND	ND	1.60 ± 0.05 a	0.19 ± 0.01 f	1.06 ± 0.01 b	0.76 ± 0.06 c	0.28 ± 0.00 e	0.48 ± 0.05 d	0.29 ± 0.01 e
Methyl caproate	E31	931	1.30 ± 0.11 e	10.87 ± 0.54 b	1.19 ± 0.04 e	6.39 ± 0.01 c	0.76 ± 0.03 ef	2.74 ± 0.09 d	ND	11.66 ± 0.83 a	0.53 ± 0 fg
Ethyl 2-methyl-2-butenoate	E32	948	ND	ND	3.71 ± 0.33 a	ND	1.53 ± 0.10 d	0.91 ± 0.01 e	2.02 ± 0.14 b	ND	1.77 ± 0.17 c
Ethyl acetoacetate	E33	957	ND	ND	0.47 ± 0.01 a	ND	0.19 ± 0.01 c	0.21 ± 0.01 b	0.13 ± 0.01 d	0.08 ± 0.01 e	0.13 ± 0.01 d
Ethyl 3-acetoxybutyrate	E34	1120	ND	ND	ND	ND	0.09 ± 0.00 c	0.35 ± 0.00 b	0.54 ± 0.03 a	ND	ND
Aldehydes											
(E)-2-Octenal	A1	1055	2.01 ± 0.01 b	2.93 ± 0.08 a	0.66 ± 0.06 c	0.35 ± 0.03 d	0.25 ± 0.02 e	ND	0.18 ± 0.02 f	0.18 ± 0.00 f	0.23 ± 0.00 ef
Benzaldehyde	A2	970	6.39 ± 0.13 d	11.86 ± 0.38 b	14.06 ± 0.01 a	6.50 ± 0.64 d	4.63 ± 0.42 e	4.78 ± 0.23 e	3.59 ± 0.3 f	3.94 ± 0.22 f	10.13 ± 0.07 c
(Z)-2-Heptenal	A3	965	1.84 ± 0.03 b	1.94 ± 0.19 b	3.24 ± 0.17 a	1.31 ± 0.13 d	1.34 ± 0.05 d	0.97 ± 0.03 e	1.51 ± 0.05 cd	1.07 ± 0.10 e	1.61 ± 0.14 c
(Z)-4-nonenal	A4	1105	0.16 ± 0.01 c	0.06 ± 0.00 d	0.17 ± 0.00 b	0.03 ± 0.00 e	ND	ND	ND	ND	0.20 ± 0.01 a
Hexanal	A5	808	56.43 ± 0.55 a	29.1 ± 2.56 b	27.47 ± 0.44 b	17.53 ± 0.18 d	16.45 ± 1.36 de	21.07 ± 1.36 c	11.63 ± 0.51 g	14.76 ± 1.29 ef	13.55 ± 0.55 fg
Nonanal	A6	1113	20.6 ± 0.74 a	15.41 ± 0.82 b	11.77 ± 0.52 c	8.42 ± 0.57 d	5.66 ± 0.11 e	10.81 ± 0.29 c	4.97 ± 0.01 e	9.14 ± 0.07 d	11.03 ± 0.92 c
trans-2-trans-6-Nonadienal	A7	1162	0.42 ± 0.01 e	0.84 ± 0.00 c	2.25 ± 0.01 a	0.61 ± 0.06 d	0.80 ± 0.03 c	0.42 ± 0.01 e	0.35 ± 0.01 f	0.65 ± 0.05 d	0.97 ± 0.01 b
2-Nonenal	A8	1169	1.01 ± 0.09 ef	1.52 ± 0.05 c	5.41 ± 0.47 a	1.15 ± 0.02 def	1.86 ± 0.18 b	0.85 ± 0.04 fg	0.59 ± 0.05 g	1.20 ± 0.07 de	1.45 ± 0.08 cd
Decanal	A9	1216	0.98 ± 0.04 c	0.75 ± 0.07 d	1.34 ± 0.02 a	0.54 ± 0.02 e	0.61 ± 0.01 e	1.03 ± 0.09 c	0.54 ± 0.04 e	1.18 ± 0.11 b	1.13 ± 0.02 b
(E; E)-2,4-nonadienal	A10	1227	ND	0.22 ± 0.02 a	0.19 ± 0 b	ND	0.18 ± 0.01 b	ND	ND	ND	ND
(Z)-2-Decenal	A11	1273	0.96 ± 0.09 a	0.44 ± 0.04 c	0.61 ± 0.01 b	0.3 ± 0.03 d	0.29 ± 0.02 d	0.43 ± 0.01 c	0.92 ± 0.03 a	0.57 ± 0.04 b	0.36 ± 0.00 d
2,4-Decadienal	A12	1305	0.38 ± 0.02 cd	0.47 ± 0.02 c	1.83 ± 0.14 a	0.52 ± 0.01 b	0.51 ± 0.04 b	0.43 ± 0.04 bcd	0.19 ± 0.01 f	0.26 ± 0.02 ef	0.34 ± 0.02 de
Undecanal	A13	1318	0.13 ± 0.01 c	ND	ND	ND	0.19 ± 0.02 a	ND	0.13 ± 0.01 c	ND	0.16 ± 0.01 b
2-Undecenal	A14	1375	0.59 ± 0.04 a	0.25 ± 0.00 d	0.45 ± 0.03 b	0.20 ± 0.01 f	0.21 ± 0.01 ef	0.24 ± 0 de	0.39 ± 0.00 c	0.36 ± 0.00 c	0.26 ± 0.02 d
Dodecanal	A15	1418	0.17 ± 0.01 e	0.25 ± 0.02 c	0.30 ± 0.00 b	0.17 ± 0.01 e	0.14 ± 0.00 f	ND	0.14 ± 0.01 f	0.23 ± 0.02 d	0.36 ± 0.01 a
2-Hexenal	A16	854	0.52 ± 0.03 h	22.03 ± 0.06 a	16.28 ± 1.06 c	17.92 ± 1.42 d	8.84 ± 0.44 d	6.10 ± 0.49 f	3.59 ± 0.11 g	ND	7.23 ± 0.37 e
Heptanal	A17	909	1.00 ± 0.02 a	0.69 ± 0.05 b	0.63 ± 0.05 c	0.44 ± 0.02 e	0.41 ± 0.04 e	0.53 ± 0.03 d	0.44 ± 0.00 e	0.57 ± 0.00 d	0.68 ± 0.03 bc
Hydrocarbons											
Tridecane, 3-methylene-	H1	1413	0.19 ± 0.01 c	0.16 ± 0.01 d	0.46 ± 0.02 a	0.18 ± 0.01 cd	0.22 ± 0.02 b	0.18 ± 0.01 cd	0.18 ± 0.02 cd	0.13 ± 0.00 e	0.10 ± 0.01 f
Cetene	H3	1604	0.16 ± 0.00 d	ND	ND	0.25 ± 0.01 b	0.29 ± 0.01 a	ND	0.22 ± 0.02 c	0.10 ± 0.01 e	ND
o-Cymene	H4	1033	0.14 ± 0.01 b	ND	0.18 ± 0.01 a	ND	0.09 ± 0.00 c	ND	ND	ND	0.17 ± 0.02 a
(1-Butylheptyl)benzene	H5	1646	0.31 ± 0.02 d	ND	0.70 ± 0.03 a	0.39 ± 0.02 b	0.32 ± 0.02 d	0.37 ± 0.03 bc	0.25 ± 0.00 e	0.27 ± 0.00 e	0.34 ± 0.03 cd
(1-Propyloctyl)benzene	H6	1657	0.25 ± 0.02 c	0.23 ± 0.01 cd	0.25 ± 0.02 c	0.13 ± 0.00 e	0.14 ± 0.00 e	0.32 ± 0.02 a	0.21 ± 0.01 d	0.21 ± 0.01 d	0.28 ± 0.01 b
3-Phenylundecane	H7	1678	0.21 ± 0.01 e	0.24 ± 0.02 d	0.32 ± 0.02 b	0.18 ± 0.01 f	0.18 ± 0.01 f	0.38 ± 0.00 a	0.29 ± 0.01 c	0.25 ± 0.02 d	0.31 ± 0.02 bc
(1-Methyldecyl)benzene	H8	1716	ND	0.32 ± 0.03 c	0.54 ± 0.04 a	0.33 ± 0.02 c	0.27 ± 0.02 d	0.5 ± 0.01 a	0.36 ± 0.01 bc	0.51 ± 0.05 a	0.40 ± 0.03 b
(1-Pentylheptyl)benzene	H9	1741	ND	0.29 ± 0.02 bc	0.27 ± 0.00 cd	0.21 ± 0.00 e	0.22 ± 0 e	0.33 ± 0.02 a	0.26 ± 0.01 d	0.30 ± 0.02 b	0.26 ± 0.03 d
(1-Butyloctyl)benzene	H10	1746	ND	0.24 ± 0.02 bc	0.22 ± 0.01 cd	0.15 ± 0 e	0.16 ± 0.02 e	0.35 ± 0.02 a	0.21 ± 0.02 d	0.25 ± 0.02 b	0.23 ± 0.02 bcd
(1-Propylnonyl)benzene	H11	1759	ND	0.22 ± 0.01 c	0.28 ± 0.01 b	0.15 ± 0.01 f	0.18 ± 0.01 e	0.31 ± 0.01 a	0.19 ± 0.01 de	0.20 ± 0.00 d	0.20 ± 0.01 d
3-Phenyldodecane	H12	1781	ND	0.33 ± 0.00 a	0.27 ± 0.02 c	0.21 ± 0.01 d	0.16 ± 0.01 e	0.30 ± 0.01 b	0.16 ± 0.01 e	0.19 ± 0.02 d	0.21 ± 0.01 d
Ethylbenzene	H13	862	0.61 ± 0.05 e	0.95 ± 0.05 b	1.34 ± 0.02 a	0.70 ± 0.01 d	0.83 ± 0.01 c	0.83 ± 0.05 c	0.67 ± 0.03 d	0.22 ± 0.00 g	0.37 ± 0.00 f
p-Xylene	H14	900	1.33 ± 0.05 d	4.39 ± 0.39 a	3.85 ± 0.22 b	3.14 ± 0.25 c	3.36 ± 0.01 c	1.58 ± 0.09 d	1.37 ± 0.03 d	0.72 ± 0.03 e	0.95 ± 0.06 e
Alcohols											
(2Z)-2-Octen-1-ol	AL1	1077	0.08 ± 0.00 c	ND	ND	ND	ND	ND	0.17 ± 0.01 b	0.09 ± 0.00 c	0.26 ± 0.02 a
Benzyl alcohol	AL2	1046	ND	ND	ND	ND	ND	ND	0.12 ± 0 c	0.16 ± 0.01 b	0.93 ± 0.03 a
Cedrol	AL3	1628	0.17 ± 0.00 c	ND	ND	ND	ND	0.21 ± 0.02 a	0.14 ± 0.00 d	0.17 ± 0.00 c	0.19 ± 0.01 b
Z-2-Hexen-1-ol	AL4	868	10.76 ± 0.12 b	ND	ND	ND	ND	1.54 ± 0.01 d	3.11 ± 0.22 c	1.97 ± 0.17 d	17.99 ± 0.85 a
Acids											
Hexanoic acid	AC1	992	8.21 ± 0.01 c	6.25 ± 0.56 d	2.91 ± 0.06 e	16.01 ± 1.23 a	1.22 ± 0.06 f	10.33 ± 0.68 b	ND	16.28 ± 1.58 a	ND
Octanoic acid	AC2	1181	0.88 ± 0.06 d	2.30 ± 0.15 a	ND	1.17 ± 0.01 c	ND	ND	ND	1.46 ± 0.01 b	ND
Ketones											
1-Octen-3-one	K1	985	0.63 ± 0.06 b	0.44 ± 0.02 c	1.08 ± 0.10 a	0.28 ± 0.02 d	0.39 ± 0.03 c	0.37 ± 0.01 c	0.41 ± 0.01 c	0.29 ± 0.02 d	0.65 ± 0.01 b
6,10-Dimethyl-5,9-undecadien-2-one	K2	1455	0.65 ± 0.04 b	0.57 ± 0.02 c	0.81 ± 0.01 a	0.32 ± 0.01 d	0.36 ± 0.01 d	0.64 ± 0.05 b	0.35 ± 0.02 d	0.6 ± 0.02 bc	0.78 ± 0.07 a
2-Heptanone	K3	896	0.28 ± 0.01 d	1.05 ± 0.05 b	ND	0.63 ± 0.06 c	ND	ND	ND	1.18 ± 0.06 a	ND
Furans											
DMMF	F1	1068	ND	9.29 ± 0.14 b	ND	7.99 ± 0.01 c	ND	10.04 ± 0.07 a	3.96 ± 0.19 e	10.28 ± 0.38 a	4.41 ± 0.08 d
DMHF	F2	1070	ND	ND	ND	ND	ND	0.33 ± 0.00 a	0.17 ± 0.02 b	0.07 ± 0.00 c	ND
trans-Linalool oxide (furanoid)	F3	1095	1.04 ± 0.06 c	1.42 ± 0.00 b	1.62 ± 0.12 a	0.81 ± 0.04 d	0.60 ± 0.06 e	1.10 ± 0.09 c	0.80 ± 0.03 d	0.90 ± 0.07 d	1.38 ± 0.12 b
Terpenoids											
Linalool	T2	1109	9.39 ± 0.19 c	2.32 ± 0.20 d	0.31 ± 0.02 f	1.26 ± 0.07 e	ND	17.08 ± 0.18 b	ND	17.51 ± 0.27 a	ND
(E)-Nerolidol	T3	1576	0.35 ± 0.01 e	3.88 ± 0.21 b	ND	1.95 ± 0.05 c	ND	34.31 ± 0.3 a	00.51 ± 0.02 de	0.65 ± 0.04 d	ND
(Z)-Nerolidol	T4	1589	ND	3.8 ± 0.17 c	ND	1.91 ± 0.09 cd	ND	34.31 ± 3.23 a	0.51 ± 0.03 d	23.17 ± 1.64 b	ND
Lactones											
γ-Dodecalactone	L1	1691	ND	0.56 ± 0.04 b	0.22 ± 0.00 d	0.57 ± 0.04 b	0.05 ± 0 f	0.93 ± 0.02 a	0.14 ± 0.00 e	0.90 ± 0.07 a	0.28 ± 0.01 c

NOTE: Refrigeration conditions: stored at 4 °C with a relative humidity (RH) of 85%. Shelf-life conditions: placed at 20 °C with a relative humidity (RH) of 80% for 2 days. Means within a column followed by different letters are significantly different (*p* < 0.05).

**Table 4:** The ROAVs for different compounds.

			Relative Odor Activity Value								
Code	Threshold (μg/Kg)	Odor Descriptor	CK 0	CK 15	MAP1 15	1-MCP 15	1-MCP + MAP1 15	CK 15 + 2	MAP1 15 + 2	1-MCP 15 + 2	1-MCP + MAP1 15 + 2
E1	1	sweet fruity pineapple waxy green banana	0	18.693	8.148	26.589	12.295	110.081	8.28	70.638	9.369
E2	55	fruity dry musty sweet wintergreen	0	0	0.018	0	0.016	0.069	0.031	0.057	0.041
E4	8	fresh green sweet fruity banana apple grassy	0	1.507	1.286	2.145	1.526	1.731	0.401	1.784	0.243
E5	2	fruity green apple banana sweet	2.452	79.835	56.139	64.518	65.724	65.014	17.22	60.767	26.25
E8	200	waxy green sweet orange aldehydic vegetable herbal	0.001	0.001	0	0.001	0	0.004	0	0.008	0
E9	2	sweet floral fruity jasmin fresh	0.095	0.199	0.127	0.366	0.192	3.291	1.299	1.56	2
E10	250	green sweet fruity apple waxy soapy	0	0.013	0	0.01	0	0	0	0.012	0
E11	15	green fruity apricot ripe banana cortex orchid fermented	0.007	0.145	0	0.12	0	0	0	0	0
E12	1.6	fruity wine waxy sweet apricot banana brandy pear	0	0	0.379	0	0.449	10.177	0.473	2.996	1.389
E13	12	green earthy mushroom herbal waxy	0	0	0	0	0.021	0.039	0	0.037	0
E15	19	green waxy apple pear banana grape	0	0.014	0	0.015	0	0	0	0.035	0
E16	19	floral rose sweet honey fruity tropical	0	0	0	0	0	0.03	0.036	0	0.027
E18	781	green natural cognac herbal waxy clean	0	0.001	0	0.001	0	0	0	0	0
E19	5	sweet waxy fruity apple grape oily brandy	0	0	0.011	0.004	0.008	0.419	0.01	0.031	0.049
E20	17	sweet balsam fruity spicy powdery berry plum	0	0.053	0.115	0.067	0.11	2.455	0.585	0.328	1.327
E23	1	fruity juicy fruit pineapple cognac	0	0	12.718	0	0	28.473	14.183	18.569	10.423
E24	43	sweet fruity estery ether pineapple ripe	0	0.007	0	0.009	0	0.003	0	0.012	0
E25	2	acrylate	0	0	0.197	0	0.199	0.047	0.78	0	0.773
E26	0.01	sharp sweet green apple fruity	0	0	377.778	0	607.692	1340.541	442.683	136.207	153.846
E27	0.15	sweet fruity banana solvent	0	5.985	21.698	18.452	38.462	39.459	78.374	37.126	34.359
E29	0.01	ethereal fruity banana pear banana apple	0	201.136	80.556	125	64.103	124.324	57.317	112.069	41.538
E31	10	fruity pineapple ether	0.103	1.235	0.055	1.141	0.097	0.37	0	2.01	0.041
E33	520	fresh fruity green apple fatty	0	0	0	0	0	0.001	0	0	0
A1	0.3	fresh cucumber fatty green herbal banana waxy green leaf	5.317	11.098	1.019	2.083	1.068	0	0.732	1.034	0.59
A2	320	strong sharp sweet bitter almond cherry	0.016	0.042	0.02	0.036	0.019	0.02	0.014	0.021	0.024
A5	4.5	fresh green fatty aldehydic grass leafy fruity sweaty	9.952	7.348	2.826	6.956	4.687	6.327	3.152	5.655	2.316
A6	1	waxy aldehydic rose fresh orris orange peel fatty peely	16.349	17.511	5.449	15.036	7.256	14.608	6.061	15.759	8.485
A7	0.5	fresh citrus green cucumber melon	0.667	1.909	2.083	2.179	2.051	1.135	0.854	2.241	1.492
A8	0.1	fatty green waxy cucumber melon	8.016	17.273	25.046	20.536	23.846	11.486	7.195	20.69	11.154
A9	1	sweet aldehydic waxy orange peel citrus floral	0.778	0.852	0.62	0.964	0.782	1.392	0.659	2.034	0.869
A10	0.062	fatty melon waxy green violet leaf cucumber tropical fruit chicken fat	0	4.032	1.419	0	3.722	0	0	0	0
A12	0.3	orange sweet fresh citrus fatty green	1.005	1.78	2.824	3.095	2.179	1.937	0.772	1.494	0.872
A13	0.03	waxy soapy floral aldehydic citrus green fatty fresh laundry	3.439	0	0	0	8.12	0	5.285	0	4.103
A15	0.1	soapy waxy aldehydic citrus green floral	1.349	2.841	1.389	3.036	1.795	0	1.707	3.966	2.769
A16	30	sweet almond fruity green leafy apple plum vegetable	0.014	0.834	0.251	1.067	0.378	0.275	0.146	0	0.185
A17	3	fresh aldehydic fatty green herbal wine-lee ozone	0.265	0.261	0.097	0.262	0.175	0.239	0.179	0.328	0.174
H13	2	pungent dry tarry	0.242	0.54	0.31	0.625	0.532	0.561	0.409	0.19	0.142
H14	530		0.002	0.009	0.003	0.011	0.008	0.004	0.003	0.002	0.001
AL2	100	floral rose phenolic balsamic	0	0	0	0	0	0	0.001	0.003	0.007
AL4	359	green cortex leafy green bean nasturtium herbal soapy aldehydic narcissus phenolic	0.024	0	0	0	0	0.006	0.011	0.009	0.039
AC1	35	sour fatty sweat cheese	0.186	0.203	0.038	0.817	0.045	0.399	0	0.802	0
AC2	1000	fatty waxy rancid oily vegetable cheesy	0.001	0.003	0	0.002	0	0	0	0.003	0
AC3	3000	waxy dirty cheese cultured dairy	0	0	0	0	0	0	0	0	0.001
K1	0.005	herbal mushroom earthy musty dirty	100	100	100	100	100	100	100	100	100
F1	0.04	sweet moldy mushroom vegetable potato burnt sugar nut skin wasabi caramellic fruity brandy	0	263.92	0	356.696	0	339.189	120.732	443.103	84.808
F2	0.03	sweet cotton candy caramel strawberry sugar	0	0	0	0	0	14.865	6.911	4.023	0
F3	60	floral	0.014	0.027	0.013	0.024	0.013	0.025	0.016	0.026	0.018
T1	10	terpene pine herbal peppery	0	0.003	0.002	0.002	0.003	0	0	0	0
T2	0.22	citrus floral sweet bois de rose woody green blueberry	33.874	11.983	0.652	10.227	0	104.914	0	137.226	0
T3	10	floral green waxy citrus woody	0.028	0.441	0	0.348	0	4.636	0.062	0.112	0
L1	0.43	fatty peach sweet metallic fruity	0	1.48	0.237	2.367	0.149	2.923	0.397	3.609	0.501

NOTE: Refrigeration conditions: stored at 4 °C with a relative humidity (RH) of 85%. Shelf-life conditions: placed at 20 °C with a relative humidity (RH) of 80% for 2 days.

**Table 5:** Effects of 1-MCP and MAP Preservation on Sensory Evaluation of Strawberry.

	Sour	Sweet	Ripe Fruit Smell	Aroma Persistence	Alcoholic Smell	Acceptability
CK0	8.67 ± 0.58 a	4.67 ± 1.15 b	6 ± 1 ab	6 ± 1 b	4 ± 0 b	7.33 ± 1.53 a
CK15	6.67 ± 0.58 b	7.67 ± 0.58 a	7.67 ± 0.58 a	7.33 ± 0.58 ab	7 ± 1.73 a	8 ± 0 a
MAP1 15	7 ± 1 a	6.67 ± 0.58 ab	7 ± 1 ab	7 ± 1 ab	7 ± 0 a	6.33 ± 0.58 a
1-MCP 15	6.33 ± 0.58 b	7 ± 1.73 ab	6.67 ± 0.58 ab	7 ± 1 ab	6.67 ± 1.53 a	7 ± 1 a
1-MCP + MAP1 15	6.33 ± 1.53 b	7.67 ± 1.53 a	7.33 ± 1.15 a	8 ± 1 a	7 ± 2 a	8.33 ± 1.15 a
CK15 + 2	5.67 ± 0.58 b	7 ± 1.73 ab	7 ± 0 ab	7 ± 0 ab	8.33 ± 1.53 a	6.67 ± 1.15 a
MAP1 15 + 2	6.33 ± 1.53 b	5.67 ± 1.53 ab	5.33 ± 1.15 b	7 ± 1 ab	8.67 ± 1.15 a	6.67 ± 1.15 a
1-MCP 15 + 2	6 ± 1 b	5.33 ± 1.53 ab	5.33 ± 1.53 b	6.33 ± 1.15 ab	9 ± 1 a	6.33 ± 1.15 a
1-MCP + MAP1 15 + 2	6.67 ± 1.53 b	7 ± 1 ab	7 ± 0 ab	7.33 ± 1.53 ab	7 ± 1 a	7 ± 1 a

NOTE: The scoring scale used in the sensory analysis was a 15-point intensity rating scale. At the same storage time point, different lowercase letters marked among different treatment groups indicate significant differences in sensory scores (*p* < 0.05).

## Data Availability

The original contributions presented in this study are included in the article/Supplementary Materials. Further inquiries can be directed to the corresponding author(s).

## Supplementary Materials

The following supporting information can be downloaded at: https://www.mdpi.com/article/10.3390/foods14172936/s1, Table S1: Comprehensive Identification Parameters of Volatile Organic Compounds in Strawberries.
