# Supplementary material for: Combined Effects of 1-MCP and Modified Atmosphere Packaging on Flavor Quality and Volatile Profile of Cold-Stored Strawberries Revealed by Untargeted GC-MS Analysis
Source: Foods. 2025 Aug 22;14(17):2936. doi: 10.3390/foods14172936 (PMC12427684; doi:10.3390/foods14172936)
Supplement: Supplementary file 1 [file foods-14-02936-s001.zip › foods-3790425-supplementary.pdf]

Table S1: Comprehensive Identification Parameters of Volatile Organic Compounds in Strawberries

VOCs	Code	CAS No.	LRI	Literature LRI	Match Factor (%)
Esters					
Ethyl hexanoate	E1	123-66-0	1008	1005-1242	92 ± 3
Ethyl benzoate	E2	93-89-0	1181	1176-1660	88 ± 2
Propyl myristate	E3	622-17-7	1872	1867-2275	86 ± 4
(Z)-3-Hexenyl acetate	E4	3681-71-8	1012	1008-1323	89 ± 3
Hexyl acetate	E5	142-92-7	1021	1018-1281	95 ± 1
2-Hexenol acetate	E6	2497-18-9	1023	1020-1335	90 ± 2
Ethyl 2-hexenoate	E7	27829-72-7	1055	1052-1380	87 ± 3
Methyl caprylate	E8	111-11-5	1133	1129-1588	85 ± 4
benzyl acetate	E9	140-11-4	1172	1168-1752	93 ± 2
Hexyl butyrate	E10	2639-63-6	1201	1198-1454	91 ± 3
2(E)-Hexenyl butanoate	E11	53398-83-7	1203	1200-1468	89 ± 2
Ethyl octanoate	E12	123-66-0	1206	1203-1435	94 ± 1
octyl acetate	E13	93-89-0	1221	1218-1393	86 ± 3
Hexyl isopentanoate	E14	622-17-7	1253	1250-1509	84 ± 4
trans-2-Hexenyl isovalerate	E15	3681-71-8	1255	1252-1524	87 ± 3
β-Phenethyl acetate	E16	142-92-7	1265	1262-1798	92 ± 2
Methyl cinnamate	E17	2497-18-9	1397	1394-1980	88 ± 3
trans-2-Hexenyl hexanoate	E18	27829-72-7	1398	1395-1621	85 ± 4
Ethyl caprinate	E19	111-11-5	1405	1402-1579	89 ± 2
Ethyl cinnamate	E20	140-11-4	1478	1475-2143	91 ± 3
2-Ethylhexyl salicylate	E21	2639-63-6	1836	1833-2224	86 ± 3
Dibutyl phthalate	E22	53398-83-7	1948	1945-2388	90 ± 2
Ethyl butanoate	E23	105-54-4	807	803-1038	95 ± 1
Isopropyl butyrate	E24	638-11-9	843	840-1064	87 ± 3
Methacrylic acid, ethyl ester	E25	97-63-2	847	844-1079	84 ± 4
Ethyl 2-methylbutanoate	E26	7452-79-1	851	848-1022	93 ± 2
Isopentyl acetate	E27	123-92-2	881	878-1120	94 ± 1
Prenyl acetate	E28	1191-16-8	920	917-1214	88 ± 3
Amyl acetate	E29	628-63-7	922	919-1165	92 ± 2
2-Methyl-2-butenyl acetate	E30	1191-16-8	930	927-1191	85 ± 4
Methyl caproate	E31	106-70-7	931	928-1293	89 ± 2
Ethyl 2-methyl-2-butenoate	E32	5464-06-4	948	945-1226	87 ± 3
Ethyl acetoacetate	E33	141-97-9	957	954-1260	86 ± 3
Ethyl 3-acetoxybutyrate	E34	141-97-9	1120	1117-1495	84 ± 4
Aldehydes					
(E)-2-Octenal	A1	2548-87-0	1055	1052-1424	91 ± 2
Benzaldehyde	A2	100-52-7	970	967-1518	93 ± 2
(Z)-2-Heptenal	A3	57266-86-1	965	962-1332	89 ± 3
(Z)-4-nonenal	A4	2277-19-2	1105	1102-1395	86 ± 3
Hexanal	A5	66-25-1	808	802-1079	96 ± 1
Nonanal	A6	124-19-6	1113	1110-1393	90 ± 3
trans-2-trans-6-Nonadienal	A7	557-48-2	1162	1159-1540	88 ± 3
2-Nonenal	A8	2463-53-8	1169	1166-1535	92 ± 2
Decanal	A9	112-31-2	1216	1213-1484	89 ± 2
(E; E)-2,4-nonadienal	A10	5910-87-2	1227	1224-1579	85 ± 4
(Z)-2-Decenal	A11	2497-25-8	1273	1270-1621	87 ± 3

2,4-Decadienal	A12	2363-88-4	1305	1302-1690	91 ± 2
Undecanal	A13	112-44-7	1318	1315-1575	86 ± 3
2-Undecenal	A14	2463-77-6	1375	1372-1664	88 ± 3
Dodecanal	A15	112-54-9	1418	1415-1720	90 ± 2
2-Hexenal	A16	6728-26-3	854	851-1160	93 ± 2
Heptanal	A17	111-71-7	909	906-1184	94 ± 1
Hydrocarbons					
Tridecane, 3-methylene-	H1	3891-98-3	1413	1410-	84 ± 4
Cetene	H3	629-73-2	1604	1601-	86 ± 3
o-Cymene	H4	527-84-4	1033	10301273	89 ± 2
(1-Butylheptyl)benzene	H5	54440-17-4	1646	1643-	85 ± 4
(1-Propyloctyl)benzene	H6	54440-18-5	1657	1654-	87 ± 3
3-Phenylundecane	H7	6731-56-0	1678	1675-	88 ± 3
(1-Methyldecyl)benzene	H8	61294-49-9	1716	1713-	86 ± 3
(1-Pentylheptyl)benzene	H9	54440-19-6	1741	1738-	84 ± 4
(1-Butyloctyl)benzene	H10	54440-20-9	1746	1743-	89 ± 2
(1-Propylnonyl)benzene	H11	54440-21-0	1759	1756-	87 ± 3
3-Phenyldodecane	H12	2719-63-9	1781	1778-	85 ± 4
Ethylbenzene	H13	100-41-4	862	859-1142	93 ± 2
p-Xylene	H14	106-42-3	900	897-1159	94 ± 1
Alcohols					
(2Z)-2-Octen-1-ol	AL1	22104-46-5	1077	1074-1615	88 ± 3
Benzyl alcohol	AL2	100-51-6	1046	1043-1869	92 ± 2
Cedrol	AL3	77-53-2	1628	1625-2170	86 ± 3
Z-2-Hexen-1-ol	AL4	928-95-0	868	865-1384	90 ± 2
Acids					
Hexanoic acid	AC1	142-62-1	992	989-1850	91 ± 3
Octanoic acid	AC2	124-07-2	1181	1178-2054	89 ± 2
Ketones					
1-Octen-3-one	K1	4312-99-6	985	982-1295	95 ± 1
6,10-Dimethyl-5,9-undecadien-2-one	K2	3796-70-1	1455	1452-1843	87 ± 3
2-Heptanone	K3	110-43-0	896	893-1180	92 ± 2
Furans					
DMMF	F1	4077-47-8	1068	1065-1529	90 ± 2
DMHF	F2	3658-77-3	1070	1067-1555	88 ± 3
trans-Linalool oxide (furanoid)	F3	34995-77-2	1095	1092-1560	86 ± 3
Terpenoids					
Linalool	T2	78-70-6	1109	1102	87 ± 4
(E)-Nerolidol	T3	142-50-1	1576	1549	85 ± 3
(Z)-Nerolidol	T4	7212-44-4	1589	1549	89 ± 2
Lactones					
γ-Dodecalactone	L1	2305-05-7	1691	1688-2254	90 ± 2

NOTE: Match Factor: Positive match factor (range: 0–100%) from the NIST14/Wiley 11 mass spectral libraries, based on full-scan mass spectrometry (m/z 33–300), presented as the mean ± standard deviation (SD) of three replicates (n = 3).
